# Pandemic scientific data sharing recommendations: examining and re-imagining pre-print servers after the end of the world-wide emergency

**DOI:** 10.1017/ash.2023.410

**Published:** 2023-08-22

**Authors:** Shira Doron, Westyn Branch-Elliman

**Affiliations:** 1 Division of Geographic Medicine and Infectious Diseases, Department of Medicine, Tufts Medical Center, Boston, MA, USA; 2 Department of Medicine, VA Boston Healthcare System, Boston, MA, USA; 3 Harvard Medical School, Boston, MA, USA

**Keywords:** pre-prints, data dissemination, public health emergency, pandemic response, data sharing, COVID-19

## Abstract

Early in the pandemic, pre-print servers sped rapid evidence sharing. A collaborative of major medical journals supported their use to ensure equitable access to scientific advancements. In the intervening three years, we have made major advancements in the prevention and treatment of COVID-19 and learned about the benefits and limitations of pre-prints as a mechanism for sharing and disseminating scientific knowledge.

Pre-prints increase attention, citations, and ultimately impact policy, often before findings are verified. Evidence suggests that pre-prints have more spin relative to peer-reviewed publications. Clinical trial findings posted on pre-print servers do not change substantially following peer-review, but other study types (e.g., modeling and observational studies) often undergo substantial revision or are never published.

Nuanced policies about sharing results are needed to balance rapid implementation of true and important advancements with accuracy. Policies recommending immediate posting of COVID-19-related research should be re-evaluated, and standards for evaluation and sharing of unverified studies should be developed. These may include specifications about what information is included in pre-prints and requirements for certain data quality standards (e.g., automated review of images and tables); requirements for code release and sharing; and limiting early postings to methods, results, and limitations sections.

Academic publishing needs to innovate and improve, but assessments of evidence quality remains a critical part of the scientific discovery and dissemination process.

## Background

Throughout the COVID-19 pandemic, dramatic press headlines about new pandemic “discoveries” related to the novel virus have driven practice carried out by clinicians, fear experienced by the public, and sometimes policy enacted by public health and governmental authorities. For the first time on a large scale, reports of scientific findings were commonly disseminated based on information gleaned from unpublished observational, laboratory, and modeling studies posted on pre-print servers without vetting or substantive quality evaluations.

Early in the pandemic, pre-print servers played an important role in speeding the rapid dissemination of evidence and allowed for widespread access to findings by the public and those without access to published articles behind a paywall. Recognizing the importance of emerging research to guide practice, a collaborative of major medical journals supported the use of pre-print servers to ensure equitable access to scientific advancements.^1^

Now that the WHO has declared the end of the COVID-19 public health emergency,^2^ should the practice of sharing and disseminating these unvetted studies continue? Will pre-prints be one of the pandemic innovations that stands the test of time? If so, how does the pre-print process need to evolve to safely support clinical advancements?

### Pre-print servers: background and evolution

**Figure 1.**
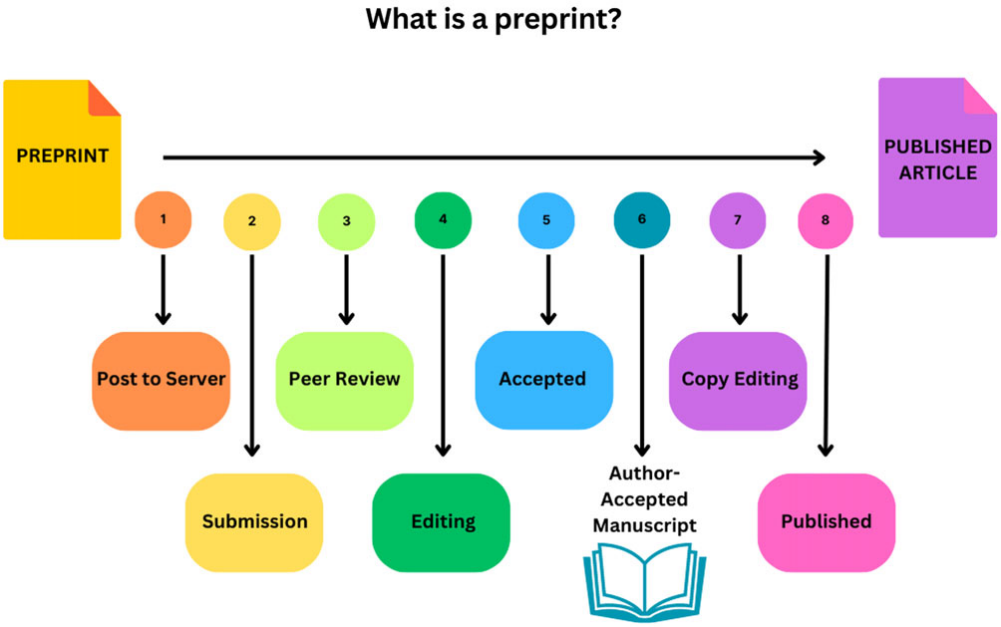
Steps in the academic publishing process.

Preprint servers are sites where authors can post their scientific papers prior to submission, review, or acceptance in a traditional journal (Fig. 1). They existed prior to the pandemic, but their use was limited, and they were not vehicles for widespread dissemination of clinical or public health studies. The first pre-print server, arXiv, was created in 1991 and covered non-medical sciences. Servers dedicated to medical and health sciences (such as medRxiv) and life sciences (such as bioRxiv) were established much later but did not rocket to popularity or serve as major information sources until the pandemic began. The most commonly used pre-print servers are not-for-profit and provide varying levels of basic screening prior to approving manuscripts (which must be original research, not case reports, editorials, or narrative reviews). Authors may submit revised versions of the same manuscript at any time, and these are tracked by the system, providing a historical record of the major changes. Some pre-print servers have affiliations with journals, particularly those that signed on to the collaborative mentioned above, facilitating direct transfer of files and saving authors time during the submission process.

### The global pandemic: a catalyst for speeding and transforming data dissemination

As the year 2020 dawned, the established mechanisms by which research findings are made available were too slow to keep pace with the needs of the healthcare and scientific communities. Even for studies that go on to be accepted, the editorial process for publication in a peer reviewed journal, which includes multiple reviews and revisions, easily takes many months, and sometimes more than a year. Clearly, with a deadly and unknown disease ravaging the globe, and new discoveries being made every day, business as usual would have been inadequate and inappropriate. The pre-print servers offered a solution to these problems.

For the first time, pre-print servers became important sources of medical information, with the papers therein amplified in traditional and non-traditional news outlets and even included in deliberations about important public health decisions, like school policy, mask mandates, and vaccine recommendations. Studies posted on pre-print servers impacted citation and alt-metric scores—two measures of research impact—and some had substantial impacts on clinical care decisions and policy without high-quality—or occasionally even verified—data to back up their claims.^3,4^

### Pre-prints: the good, the bad, the ugly

#### The good

There are a number of benefits associated with posting manuscripts to pre-print servers prior to publication, including (1) the findings are immediately widely available for viewing and citing and, unlike many journal sites, without a pay wall; (2) feedback received from the research community and (unlike what scientists might otherwise get when they present their work at conferences) the general public can be used to improve the quality of the manuscript prior to submission to journals and/or during the revision process; (3) leveraging the timestamp on the posting can avoid disputes about originality of research or the timing of findings compared to that of other researchers, and (4) because the sites include information about version numbers and revisions, they can serve as a mechanism for promoting transparency and accountability. As noted above, authors benefit from increased visibility and alt-metric scores, which are a measure of publication impact. Researchers can also benefit by using pre-print servers as a mechanism for sharing preliminary research findings used to support grant proposals.

#### The bad

Although dissemination of COVID-19-related research through pre-prints was widely accepted and adopted before major therapeutic and preventative milestones were reached, the approach has many limitations, which have important implications for public health and science communication. Quality checks are limited, and there is no standardized process for ensuring accuracy. Among clinical trials that are ultimately published, findings do not change substantially following peer-review, although presentation of results tends to be more complete and with less perceived spin.^5,6^ However, the same cannot be said for other study designs, or for clinical trial results that never go on to get published. A recent study found that one in five randomized controlled trials remained unpublished 12 months after posting, and those that were not published were less complete and more highly spun than those that had undergone the peer review process and were ultimately published in a journal.^7^ Findings are even more stark for other study designs. Although published literature on the topic is inconsistent, estimates are that only approximately two-thirds of pre-prints go on to get published^8^ of these, about 17% undergo major changes during the revision process,^9^ meaning that almost *half* of the papers posted to pre-print servers are either changed substantially or never make it through peer-review.

#### The ugly

Anchoring bias is a well-described concept in psychology and refers to the strong tendency of humans to weight the first piece of information they receive more heavily than future information.^10^ Disclaimers are insufficient to overcome this basic human instinct. Many early reports are later debunked by better quality research, and promising interventions tested in laboratory or modeling settings often are found to be ineffective when tested in human populations. This tension is amplified because laboratory, modeling, and simulation studies are inherently faster to conduct. Human subjects are protected, drugs are regulated before they can be tested, and recruitment, outcomes assessments, and data analysis all take time.

Once data are available, findings and early presentations are widely shared on social and traditional media outlets, impacting perceptions and, at times, policy-making decisions. Pre-print servers do not create these information dissemination problems, but they do accelerate them. Thus, even updated policy about the use of pre-print servers is unlikely to be sufficient on its own to change the problems posed by factors inherent to the speed of the scientific discovery process or to how data are shared and discussed in public forums.

### Pre-prints: empirical evidence of impact

These concerns are not just theoretical but supported by empirical evidence. Likely driven in part by anchoring bias, despite these well-documented limitations of different study designs and analyses, by the time contradictory or higher-quality evidence became available, the first report has already made its lasting mark. For example, a study by one of the authors (WBE) measured the speed and scope of COVID-19 prescribing practices in the Veterans Health System and found that practice patterns changed rapidly after data release via pre-print servers and press releases, with limited additional change after peer-reviewed publication.^11^ Effects were strongest early in the pandemic, when there were few treatment options, and waned as time went on. Studies on the efficacy of the bivalent COVID-19 vaccine in human subjects have been presented at regulatory meetings before peer review, allowing for expedited authorization and guideline development, potentially benefiting society by reducing delays.^12,13^ On the other hand, even after a pre-print purportedly demonstrating efficacy was withdrawn from the server due to serious ethical infractions,^14^ ivermectin has continued to be prescribed despite a preponderance of evidence demonstrating lack of efficacy.^15,16^ Other examples of practice changes driven by early studies with major limitations include “gaiter-gate”.^17,18^ Despite rapidly available contradictory evidence, the damage was done. Gaiters were widely banned in schools based on an early report that they were inferior to other cloth masks and subsequent dissemination via traditional and social media sources.^19,20^ In another example, a research group using publicly available CDC data published a pre-print in May 2022 that calculated that COVID-19 was the 4^th^ highest cause of death in children under the age of 19.^21^ This analysis was shared in both the FDA’s VRBPAC meeting and the CDC’s ACIP meeting to inform discussion about COVID-19 vaccination policy for children. Findings were also widely shared in traditional and social media outlets. A revised version of the pre-print,^22^ however, listed COVID-19 as the 7^th^ highest cause of death, and in the final publication, COVID-19 was calculated to be the 8^th^ highest cause of death.^23^ During the revision process, the estimated crude mortality rate of COVID-19 for children changed quite dramatically, from 7.2 down to 1.0 per 100,000 from the first version of the pre-print to the final, peer-reviewed published version of the manuscript.

### Evolving pre-prints to meet current needs and conditions

Recognizing the limitations of pre-prints, their benefits, and their real-world impacts raises important questions about how the use of pre-print servers for disseminating medical evidence should evolve as we move into the next phase of living with COVID-19 (Table 1).^24^ First, given the aforementioned data demonstrating that one of the biggest impacts of peer-review is to change framing and reduce spin, a pre-print policy change that could be trialed is presentation of methods, results, and limitations sections *only* without background and discussion sections, which are fundamentally more prone to personal opinion and political viewpoints. Another possibility is for pre-print servers to be re-organized, such that limitations are a required element that are presented before the results section. This would ensure that limitations are highlighted so that the public can view and comment on them; reading limitations first might also change the reader’s perceptions about the implications of the results, partially (if not completely) addressing anchoring bias tendencies. Additional requirements for quality checks and release of underlying methodology (e.g., by requiring code to be shared with the release, or publication of models so that others can review) would also be options for improving pre-print transparency and evaluations. Development and application of advanced technology to identify falsified images, calculation errors, and plagiarism are additional considerations that should be considered, supported, and evaluated to improve evidence quality.

**Table 1.** Pre-prints: benefits and downsides relative to traditional academic publishing

Benefits	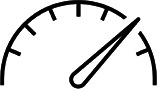	Rapid Release
	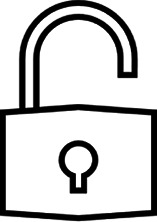	Equitable Access
	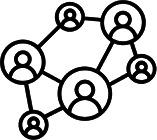	Crowd-Sourcing Research Feedback
	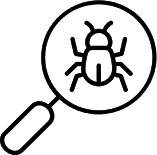	Ability to View Timestamp, History and Updates
Downsides	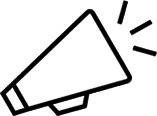	Politics and Spin
	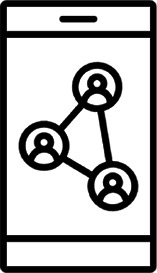	Excessive Influence
	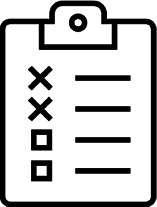	Many Never Pass Quality-Checks and Review

### Evolving existing academic publishing and data sharing

The influence of pre-prints has not occurred in a vacuum. They are one piece of the puzzle and changes at many levels of the system are needed to improve data dissemination. Before pre-prints are widely shared by influencers, covered by the media, and communicated to the public as “truth,” uncertainty about the study’s eventual conclusions and implications of the work needs to be acknowledged by those who are responsible for sharing and spreading the information.

Although pre-prints in their current iteration may not be the ultimate solution, innovation in medical publishing and dissemination of research findings is badly needed. Academic publishing is strongly biased toward publishing papers—and questioning findings less—if authored by those who are already “famous.”^25^ Peer review is slow, biased, and often does not lead to major changes even when they are needed,^26^ as occurred with the Surgisphere debacle, in which the underlying data supporting the harms of hydroxychloroquine for COVID-19 treatment could not be verified.^27^ For topics of critical public health importance, rapid review pathways should be adopted to ensure that data from high-quality randomized controlled trials are quickly available to inform clinical care decisions. Peer-reviewed manuscripts in traditional journals should be readily available and accessible without a subscription, and access needs to be expanded; Plan S in Europe^28^ and the White House Office of Science and Technology policy on free, immediate, and equitable access are important policy changes aimed to address these barriers.^29^ Open access should be the standard, and The Journal of the American Medical Association took big steps in this direction in December 2022.^30^ The National Library of Medicine could require indexed journals to, at a minimum, make methods, results, and limitations openly accessible to the public and set quality standards to encourage expansion of open-access models.

The financial incentives of academic publishing also inherently create challenges. Academic journals rely on the donated time of unpaid reviewers, which may limit review quality. Journals requesting peer-review are asking for time and expertise—things that outside of academia are always compensated. The practice of donated reviews may be defensible for society journals that do not have publication fees and therefore providing an expert review free-of-charge is a *quid pro quo*. However, given the rise of for-profit journals and major publishing enterprises, some of which boast profit margins higher than those of Apple or Google,^31^ due in part to the free labor and expertise provided by reviewers—the compensation structure, or lack thereof, needs reevaluation. However, despite all of these caveats and limitations, recent data suggest that the speed and quality of peer review may actually have increased during the pandemic.^32,33^

### Next steps

What is the best path forward? Evidence of quality and our willingness to act upon findings need to be balanced (Table 2). Data from pivotal clinical trials should be reviewed and released rapidly, so that clinicians and policy makers can translate evidence into care immediately; recently released data on the harms associated with corticosteroids for inpatients without severe COVID-19 is an example that should immediately change practice.^34^ For other studies, speed and content quality could be balanced by rapid posting of accepted, but not publication proofed, articles.

**Table 2.** Framework for considering data release and sharing

Speed	Clinical trials prioritized for rapid review Laboratory, observational, and modeling studies undergo traditional vetting processes
Quality	Develop systems for assessing quality (e.g., automated systems for reviewing images and text to confirm authenticity) Automated solutions to review tables, etc. for accuracy and reliability Requirements for releasing underlying protocols or code used to conduct the research
Anchoring	Evaluation of novelty of findings and potential public health impact Limitations specified upfront
Spin	Prereviewed releases focus on methods, results and limitations Background sections and discussion sections not included Consider open peer-review processes
Equity	Key study findings universally available free of charge Develop new models for traditional publications (e.g., publish methods, results, and limitations) even if full open-access model is not supported
Impact	Evidence standards for traditional media release Requirements for quality checks prior to sharing Guidelines about completeness of data and methods prior to publication and sharing

Mainstream and social media outlets should wait for observational and laboratory-based research to undergo a vetting process. Denise-Marie Ordway wrote in The Journalist’s Resource in April 2020 that there are 6 things journalists must know before covering biomedical research pre-prints about COVID-19:^35^ (1) pre-prints can be dangerous if doctors change their practice based on the results; (2) pre-prints are not peer reviewed, leaving them without opportunity for other experts to catch errors, argue with the authors’ interpretation of findings, or request additional data or analyses; (3) best practice dictates that reporters should be explicit that findings reported in pre-prints are preliminary and, ideally, the opinion of an expert should be included to counter inappropriate conclusions; (4) pre-prints are best covered by experienced science journalists; (5) journalists should check with experts to determine whether findings from a pre-print are even worthy of coverage; (6) pre-prints are sometimes withdrawn, particularly after feedback from the scientific community alerts authors to flaws in their methodology or conclusions.^34^

### Conclusions

Policies recommending immediate posting of COVID-19-related research should be re-considered—case reports, observational, and modeling studies should be made publicly and freely available after an evaluation process and with quality standards. Academic publishing needs to innovate and improve—but evidence of quality assessments remain a critical part of the scientific discovery process. We need to take a step back and digest the data before it is disseminated and implemented. To achieve real improvement, we also need to re-think how and when information is shared.
